# Long non-coding RNAs and JAK/STAT signaling pathway regulation in colorectal cancer development

**DOI:** 10.3389/fgene.2023.1297093

**Published:** 2023-11-29

**Authors:** Abdolmajid Ghasemian, Hadeel A. Omear, Yaser Mansoori, Pardis Mansouri, Xinpei Deng, Farzaneh Darbeheshti, Elham Zarenezhad, Maryam Kohansal, Babak Pezeshki, Zhangling Wang, Hailin Tang

**Affiliations:** ^1^ Noncommunicable Diseases Research Center, Fasa University of Medical Sciences, Fasa, Iran; ^2^ College of Science, University of Tikrit University, Tikrit, Iraq; ^3^ State Key Laboratory of Oncology in South China, Sun Yat-sen University Cancer Center, Guangdong Provincial Clinical Research Center for Cancer, Guangzhou, China

**Keywords:** long non-coding RNAs, colorectal cancer, Janus kinase/signal transducer and activator of transcription, signaling pathways, regulation

## Abstract

Colorectal cancer (CRC) is one of the main fatal cancers. Cell signaling such as Janus kinase/signal transducer and activator of transcription (JAK/STAT) signaling substantially influences the process of gene expression and cell growth. Long non-coding RNAs (lncRNAs) play regulatory roles in cell signaling, cell proliferation, and cancer fate. Hence, lncRNAs can be considered biomarkers in cancers. The inhibitory or activating effects of different lncRNAs on the JAK/STAT pathway regulate cancer cell proliferation or tumor suppression. Additionally, lncRNAs regulate immune responses which play a role in immunotherapy. Mechanisms of lncRNAs in CRC via JAK/STAT regulation mainly include cell proliferation, invasion, metastasis, apoptosis, adhesion, and control of inflammation. More profound findings are warranted to specifically target the lncRNAs in terms of activation or suppression in hindering CRC cell proliferation. Here, to understand the lncRNA cross-talk in CRC through the JAK/STAT signaling pathway, we collected the related *in vitro* and *in vivo* data. Future insights may pave the way for the development of novel diagnostic tools, therapeutic interventions, and personalized treatment strategies for CRC patients.

## Background

Colorectal cancer (CRC) is one of the leading causes of cancer-related death, with a survival rate of nearly 50% (Misale et al., 2012; Hugen et al., 2016). Cell signaling pathways such as Hippo, Janus kinase signal transducer and activator of transcription (JAK/STAT), NOTCH, SHH/GLI, TGF/SMAD, and Wnt/β-catenin play substantial roles in cancer initiation and progression (Takebe et al., 2015; Park et al., 2020; Zou et al., 2020; Zhou et al., 2022). These pathways contribute to the epithelium maintenance and stem cell multiplication and differentiation. STAT signaling participates in numerous cellular processes such as immune regulation and cancer progression (Aggarwal et al., 2009; Yu et al., 2014). STATs including STAT1–6 may exacerbate cell growth and cancer development (STAT3 and STAT5) and regulate the anti-tumor immune responses for tumor control (STAT1 and STAT2) (Owen et al., 2019; O'Shea et al., 2015). For instance, cytokines such as IL-2, IL-15, IL-12, INF-α/β, and INF-γ have been associated with the STATs (Heinrich et al., 2003; Morris et al., 2018). In protumor conditions, Th2, Tregs, Th17, and myeloid-derived suppressor cells (MDSCs) secrete IL-1, IL-10, IL-17, and TGF-β through STAT3 and STAT5 signaling, which precludes immune function in the tumor microenvironment (TME) (Seif et al., 2017; Yoshimura et al., 2018; Kang et al., 2019). STATs 1, 2, 3, and 5 enter the nucleus and control the interferon gene transcription. STAT1 and STAT4 mainly contribute to cytokine production and cell-specific activity, which results in the anti-tumor effects of immune cells (MHC-I and checkpoint inhibitor upregulation) (Erdogan et al., 2022). The JAK/STAT pathway can be regulated in several cancers by microRNAs, circular RNAs, and long non-coding RNAs (lncRNAs) (Yan et al., 2020).

Transcription of 85% of mammalian genomes infers the critical role of RNAs in biological systems. lncRNAs, comprising >200 bp, have wide biological activities such as transcriptional or post-transcriptional functions and interference with RNA processing, thereby promoting or inhibiting tumorigenesis (Yang et al., 2014; Chi et al., 2019; Wu W. et al., 2022). These RNAs lack open reading frames (ORFs) and interact with proteins, miRNAs, and other lncRNAs. Mechanisms of lncRNA functions include natural antisense transcripts (NATs), chromatin interactions and remodeling, and ceRNAs (Beermann et al., 2016). Numerous lncRNAs are upregulated or downregulated in cancers like CRC, influencing cell signaling pathways (He and Wu, 2023). Genetic or epigenetic factors underlie the lncRNA expression varieties in cancer (Shaker et al., 2017). lncRNAs KCNQ1OT1, HOX transcript antisense RNA (HOTAIR), and metastasis-associated lung adenocarcinoma transcript 1 (MALAT1) have been dysregulated in colorectal cancer (Kogo et al., 2011; Xu et al., 2011; Niinuma et al., 2012). The lncRNA HOTAIR/miR-326/FUT6 axis facilitates the development of CRC via PI3K/AKT/mTOR signaling (Pan et al., 2019). lncRNA-H19 regulates miR-29b-3p and leads to CRC progression (Ding et al., 2018).

JAK/STAT activation or regulation by lncRNAs has been unveiled in CRC (ITIH4-AS1), chronic myeloid leukemia (MEG3) (Li et al., 2018), gastric cancer (LINC00691), osteosarcoma (lncRNA 135528), and non-small-cell lung cancer cells (PART1). In the CRC cells/tissues, the JAK/STAT pathway has been regulated by RP11-468E2.5, LINC01116, AB073614, SUMO1P3, HAND2-AS1, MIR100HG, TRG-AS1, PCED1B-AS1, FAM30A, AL365361.1, AC090559.1, LINC01094, LINC00346, TPT1-AS1, and HOXA11-AS. It was observed that the downregulation of the RE1-silencing transcription (REST) factor led to the upregulation of ITIH4-AS1 and contributed to CRC via FUS-mediated JAK/STAT3 signaling activation (Liang et al., 2019; Ming et al., 2021). In the current review, the available data on the lncRNA-mediated JAK/STAT pathway in CRC are analyzed to indicate how pivotal lncRNAs could be in CRC progression and management.

## JAK/STAT and colorectal cancer

The sensing, cytokine receptor binding, and phosphorylation of the JAK/STAT pathway lead to related gene regulation. The activation of STATs results in the transcriptional regulation of immune responses, apoptosis, inflammation, proliferation, and angiogenesis. Genetic mutations in the JAK-STAT pathway lead to aberrant activation, even without the cytokine induction, which results in persistent activation and tumorigenesis. For instance, persistent overexpression of STAT5 occurs in the neoplasia stage. It was revealed that circular RNA circSPARC promotes the invasion of CRC cells via JAK/STAT pathway regulation (Wang J. et al., 2021). A previous study showed that activating transcription factor 1 (ATF1) was associated with CRC progression via regulating the JAK/STAT, TNF, and Wnt pathways. ATF1 was also associated with the two lncRNAs PVT1 and CCAT1, which played a role in the exacerbation of CRC (Wang H. et al., 2021). CCAT5 also upregulates STAT3 in CRC (Wang et al., 2020). The ADAM10-JAK-STAT signaling pathway can also be regulated by miR-365a-3p, thus inhibiting CRC cell proliferation (Hong et al., 2020). STAT3 also mediates the activation and infiltration of tumor-specific T cells. A bibliometric analysis deciphered a myriad of lncRNAs associated with CRC progression, invasion, and metastasis (He and Wu, 2023). The downregulation of STAT3 by miR-34a inhibits CRC cell metastasis. In addition, interactions between CASC2, miR-18a, PIAS3 (an mRNA molecule), and the STAT3 signaling pathway caused CRC cell multiplication and tumor development (Huang et al., 2016a).

## Long non-coding RNA levels change in cancer and cancer therapy

**FIGURE 1:**
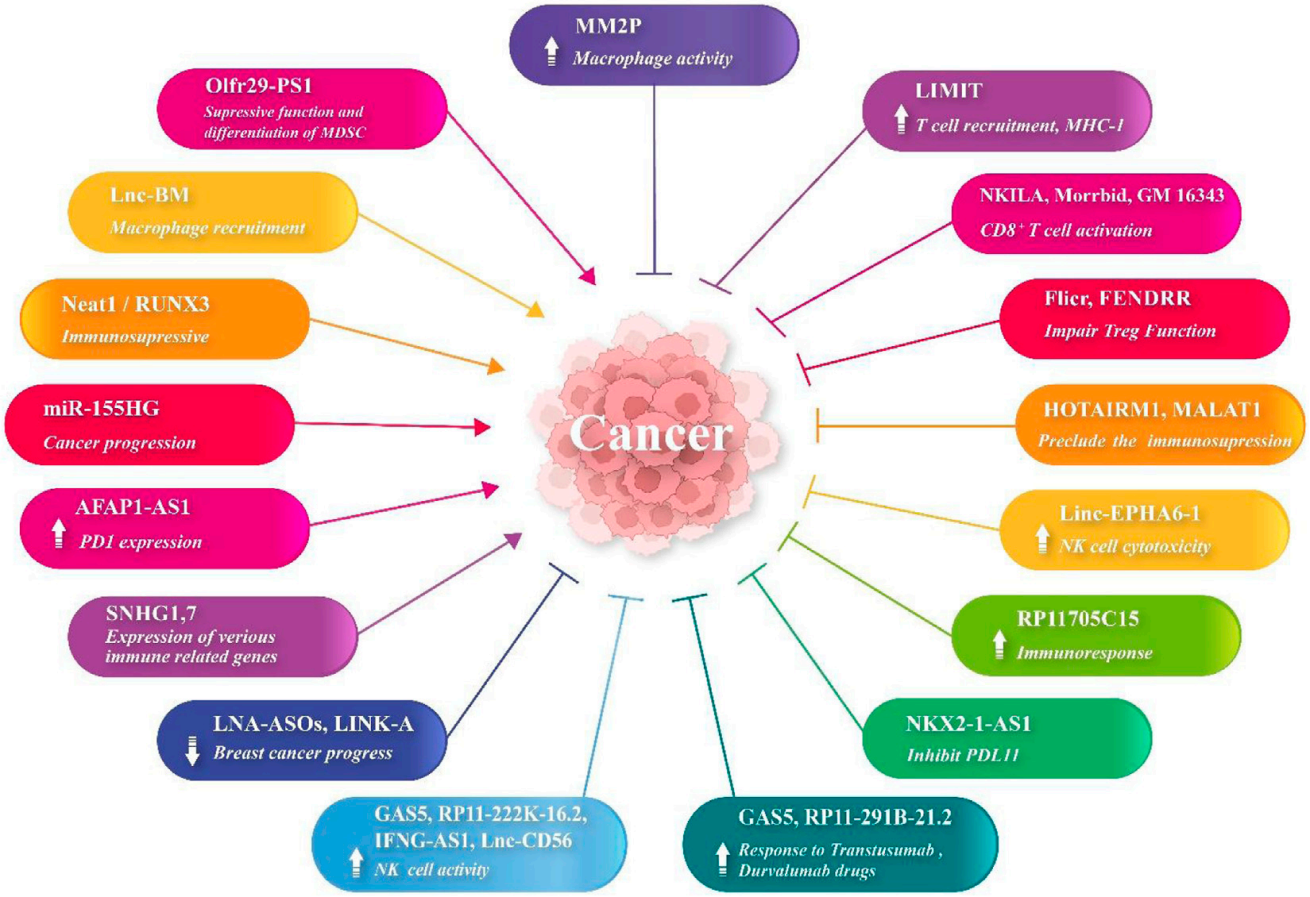
lncRNA expression changes in cancers.

lncRNAs are involved in cancer cell multiplication and metastasis through a myriad of cellular processes and common signaling pathways, where lncRNAs can bind to some specific DNA or protein complexes (Chen et al., 2021). These include phosphatidylinositol-3-kinase (PI3K), P53, KRAS, epidermal growth factor receptor (EGFR), Wnt/β catenin, epithelial–mesenchymal transition (EMT), and TGF-β signaling pathways (Lin et al., 2021). The lncRNA regulator of reprogramming (*lnc*-ROR) modulates the exacerbation of various cancerous conditions (Lulli et al., 2022; Tabarestani et al., 2022). The TME metabolic conditions also regulate the expression of lncRNAs, which leads to the expression of various enzymes and signaling pathways. A previous study unveiled that the aberrant expression of LINC00152 develops CRC conditions (Ou J. et al., 2021; Li S. et al., 2022). lncRNA HOXB-AS3 encodes a peptide which inhibits CRC cell growth via metabolic regulation (Huang et al., 2017). lncRNA SNHGS mediates CRC cell resistance to oxaliplatin via deregulation of STAU1 (Chen F. et al., 2022). Moreover, expression levels of lncRNA RP11-462C24.1 regulate PI3K/AKT and HSP70 signaling in the CRC tissues (Zhang H. et al., 2021). lncRNA ADAMTS9-AS2 affects the development of various cancers, such as the tumor metastasis of glioma cells and gastric and lung cancers, while lncRNA DANCR develops tumorigenesis by osteosarcoma via repression of miR-33a-5p (Yao et al., 2014; Liu et al., 2018; Ren et al., 2020). lncRNA LINC01116 can target miR-520a-3p and VEGFA and arrest cell proliferation and preclude brain and osteosarcoma tumorigenesis [lnc3 introduction (Ye et al., 2020)]. It also regulates miR-9-5p-STMN1 and EZH2-regulated TPM1, hence promoting CRC development (Bi et al., 2020a; Liang et al., 2021a). lncRNA OTUD6B-AS1 was associated with thyroid and renal carcinoma inhibition via regulation of Wnt/β-catenin signaling (Wang et al., 2019). Additionally, its overexpression has reduced CRC cell proliferation mainly via regulation of miR-21-5p and proline-rich nuclear receptor coactivator 2 (PNRC2) (Cai et al., 2021; Wang W. et al., 2021). MEG3 is an lncRNA, which inhibits carcinogenesis through the regulation of JAK/STAT-activating miR-9 (He et al., 2017; Moradi et al., 2019). Other cell-proliferative and metastatic lncRNAs include PCGEM1, SNHG15, POU3F3, PVT1, MALAT1, TUG1, urothelial carcinoma-associated 1 (UCA1), and PCAT-1 (Jin et al., 2020; Zhu and Zhu, 2022). Tumor-suppressive lncRNAs include growth arrest-specific transcript 5 (GAS5), MEG3, DGCR5, PTENP1, and NKILA (Yu and Hann, 2019). Immune escape is mediated by MALAT1, UCA1, and SNHG1 (Liu et al., 2020). Angiogenesis is mediated by MEG3, TUG1, and CASC3. Some lncRNAs cause metastasis (CCAT2, H19, and NKILA), cell stemness (ASAP-1-IT-1, BCAR4, and ARSR), drug resistance (PVT1, HOTAIR, and AX747207), and DNA damage (DDSR1, ANRIL, and NEAT1). Moreover, lncRNAs regulate miRNAs which regulate cell signaling and cancer progression. The regulation of intestinal epithelium maintenance by lncRNAs has been documented (Xiao et al., 2019) (Figure 1).

Low immune response and immune evasion lead to cancer cell multiplication, invasion, and metastasis. Although various lncRNAs (such as Neat1 and RUNX3) (Pandey et al., 2022) have participated in immunosuppression in cancers, some of them have exhibited immunostimulatory properties (Eptaminitaki et al., 2021). lncRNAs are of significance in the multiplication and activation of CD4^+^ and CD8^+^ T cells and NK cells (Li et al., 2021). MHC-I and immunogenicity of tumor (LIMIT) is an lncRNA, which provokes the expression of the MHC-I gene via detachment of heat shock factor-1 (HSF1) and HSP90 proteins. RNA-guided CRISPR activation results in MHC-I overexpression, T-cell recruitment, and checkpoint blockade response (Li et al., 2021). lncRNA AFAP1-AS1 increases PD-1 molecule expression in nasopharyngeal carcinoma (Tang et al., 2017). lncRNA Olfr29-ps1 targets miR-214-3p, which decreases MDSC activity and differentiation mediated by MyD88 (Shang et al., 2019). lncRNA RP11705C15 provokes efficient immune responses against NSCLC cells following anti PD-1 immunotherapy (Xu et al., 2018). lncRNA MIR-155HG is also associated with the overall survival (OS) of patients suffering from various cancers (Li et al., 2016). lncRNA Flicr impairs the Treg functions and, hence, enhances the immune responses. CD8^+^ T-cell activation is mediated by NKILA, Morrbid, and GM16343. In addition, MM2P enhances macrophage activation. HOTAIRM1 and MALAT1 preclude MDSC-mediated immunosuppression. GAS5, RP11-222K-16.2, IFNG-AS1, and lnc-CD56 enhance NK cell activity. In hepatocellular carcinoma, FENDRR hinders the activity and multiplication of Tregs. lnc-BM regulates macrophage recruitment into the brain and HCC TME. In NSCLC, linc EPHA6-1 enhances NK cell cytotoxicity. In lung adenocarcinoma, NKX2-1-AS1 inhibits PDL-1 expression. Furthermore, lncRNAs GAS5 and RP11-291B-21.2 enhance the responses to trastuzumab and durvalumab, respectively. An *in silico* study demonstrated that high expression levels of two lncRNAs SNHG1 and SNHG7 (using GEO analysis and GSEA) in HCC tissue were associated with the expression of various immune-related genes. These genes were mainly related to infiltration and checkpoint inhibitors (Chen E. et al., 2022). The combination of locked nucleic acid (LNA)-ASOs and LINK-A lncRNA has suppressed breast cancer progression (Hu Q. et al., 2019). Moreover, the incorporation of lncRNA into CAR T cells has played the role of an adjuvant in the regulation of T-cell apoptosis and suppressed tumor immune evasion and enhanced the immunotherapy (Huang et al., 2018).

**FIGURE 2:**
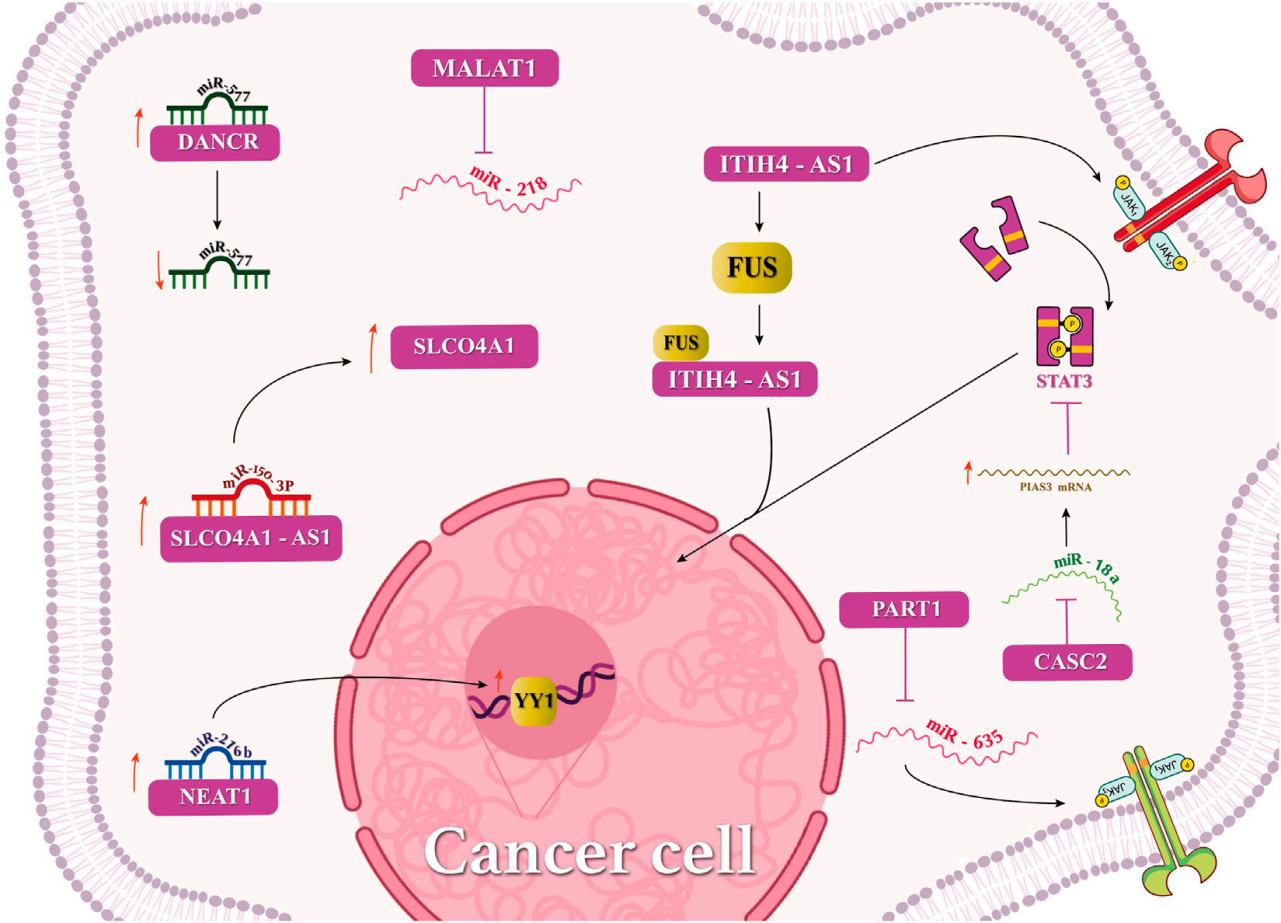
Mechanisms of lncRNAs in cellular pathway regulation in cancers.

Interactions of lncRNAs with miRNAs, mRNAs, or circular RNAs have been investigated in CRC (Figure 2), highlighting meaningful effects in the disease fate. lncRNA MALAT1 interacts with miRNA-218, which accelerates metastasis and EMT. Additionally, MALAT1 suppresses miR-106-5p and promotes the SLAIN2 expression. lncRNA CYTOR and LINC00707 binding to miR-3679-5p and miR-206, respectively, has promoted the development of CRC. Furthermore, lncRNAs DANCR and UICLM have sponged miR-577 and miR-215, respectively, causing the metastasis of CRC. CCAT2 has modulated the expression of miR-145, leading to the mutation inhibition of CRC cells (Niu et al., 2021; Tan et al., 2021). LAMTOR5 or LAMTOR5-AS1, a proposed biomarker in CRC, which is highly expressed in elderly patients, has sponged hsa-miR-20a-5p and hsa-miR-let-7b-3p (Zaniani et al., 2021). LAMTOR5 was not a suitable biomarker for CRC. An *in silico* study revealed that lncRNAs SNHG5, GATA2-AS1, and H19 with various miRNAs facilitated CRC (Shaath et al., 2021). The high expression of LINC00963 but not miR-1281 has been reported in CRC, and their interaction has caused the downregulation of miRNA-1281. lncRNA NEAT1 has sponged miRNA-216b and activated YIN-YANG-1 (YY1), thus accelerating CRC tumorigenesis (Zhu et al., 2022). Interactions of SLCO4A1, miR-150-3p, and SLCO4A1-AS1 have been investigated in colon cancer tissues. SLCO4A1-AS1 binds to miR-150-3p and enhances the expression of SLCO4A1, which increases CRC tumorigenicity (Wu et al., 2021).

The lncRNA–miRNA interaction regulates several cancers. For instance, the binding of lncRNA MALAT1 and miR-200c leads to the progression to EMT. The binding of lncRNAs Sox2ot and XIST to miR-200a and miR-429, respectively, exerts similar effects. lncRNA PCED1B-AS1 and each of miR-411-3p and miR-29b also mediate such effects. THAP9-AS1 has sponged miRNA-484 and has led to Hippo pathway regulation in cancer. DANCR has sponged the tumor-suppressive miRNAs 33b, 135a-5p, 613, 34c, 34a-5p, 149,496, 1972, and 335-5p to regulate cancers via several signaling pathways (Farooqi et al., 2021). Regarding CRC (Yang et al., 2021), lncRNA XIST regulates miRNA-200b-3p, which hinders the ZEB1 function and inhibits metastasis and EMT. lncRNA MALAT1 sponges miR-126-5p and prohibits VEGFA in the angiogenesis process (Sun et al., 2019). lncRNA HOTAIR binds to miR-218, which may control NF-KB signaling and resistance to 5-fluorouracil or facilitate cancer development (Li et al., 2017; Hu X. et al., 2019; Wei et al., 2020). lncRNA HAGLROS binds to miR-100 and controls ATG5 in autophagy, which activates the PI3K/AKT and mTOR pathways. In the stemness process, lncRNA BCAR4 regulates miR-665 and controls STAT3, which, in turn, regulates the expression of NANOG, OCT4, SOX2, CD44, CD133, and Lgr5 proteins. Several other miRNAs, such as miR-137 (by SNHG1), miR-181a-5p (by ZEB1-AS1), miR-200a, miR-29b-3p, and miR-138 (by the H19 oncogene), miR-1271 (by HCG18), miR-204 (by PlncRNA-1), miR-203a-3p and miR-214 (by HOTAIR), miR-34a and miR-200b-3p (by XIST), miR-129-5p (by HIF1A-AS2), miR-497 (by TTN-AS1), miR-203 (by B3GALT5-AS1), miR-215 (UICLM), miR-101-3p (SNHG6), miR-489/TWIST1 (by CHRF), miR-600/KIAA1199 (by TUG1), and miR-150-5p (by ZFAS1), have been reported (Huang et al., 2016a; Bian et al., 2016; Wu et al., 2019; Chodary Khameneh et al., 2022).

## JAK/STAT pathway targeting in immunotherapies

The enhanced expression of cytokines and their receptors mainly result in the aberrant regulation of JAK1/2, STAT1, STAT3, STAT5, and STAT6, causing inflammation and cancer development, cancer recurrence, and decreased overall survival. JAK1 and STAT3 are necessary for T-cell activation. Hence, the impairment in the system affects efficient cancer cell combating and even leads to T-cell lymphoma (Waldmann and Chen, 2017). INF-JAK/STAT pathway targeting can provide efficient outcomes in immunotherapy and radiotherapy, considering sufficient strength and duration of treatment. JAK1 signaling has participated in PDL-1-mediated melanoma immunotherapy (Luo et al., 2018; Shi and Bonner, 2021). Moreover, JAK/STAT targeting, particularly of combination therapy, is promising for glioblastoma treatment (Ou A. et al., 2021; Shi and Bonner, 2021). STAT3 activation suppresses immune cells and targets interleukin-6 (IL-6), which activates MDSCs and shifts Th1/Th2 balance to Th2 type. Therefore, JAK/STAT inhibitors are promising for cancer therapy (Sabaawy and Zeeshan, 2021). The suppression of STAT5, an activator of CD4+/CD25+ Tregs, can activate immune cells such as NK cells (Gotthardt et al., 2016). Targeting JAK/STAT signals leads to the inhibition of chronic inflammation, anti-tumor cell suppression, and more effective cancer therapy (Sabaawy et al., 2021). The human microbiota may affect the expression of lncRNAs, such as HOTAIR, LINC00491, KCNQ1OT1, and LINC00355, by CRC cells (Yang et al., 2020; Khodaii et al., 2022). RPS6, PMAIP1, BCL2, and FAM129A genes were associated with immune cell infiltration (Tan et al., 2021).

## Long non-coding RNAs and JAK/STAT regulation

lncRNA 135528 can regulate the JAK/STAT pathway and then CXCL10, which precludes tumorigenesis (Wang K. et al., 2021). lncRNA HOTAIR leads to liver cancer progression via SETD2 regulation. In addition, lncRNA HOTTIP leads to pancreatic cancer progression by the regulation of HOXA9 expression. lncRNA XIST/miR-200c also regulates bladder cancer. lncRNA PART1 provokes the JAK‐STAT signaling pathway, which facilitates the non-small-cell lung cancer cell proliferation *in vitro* and *in vivo*. The main regulation mechanisms and results of the JAK/STAT signaling pathway include FUS-dependent JAK/STAT3 pathway activation (ITIH4-AS1), miR-18a/STAT3 sponging (CASC2), apoptosis induction via STAT5 and STAT6 (RP11-468E2.5), apoptosis induction via targeting STAT5/6 (RP11-468E2.5), CRC development via miR-9-5p/STMN1 and IL-6R regulation (lncRNA LINC01116), JAK/STAT3 pathway downregulation, expression of E-cadherin and occludin (lncRNA AB073614), cell proliferation and inhibition of apoptosis, JAK1/STAT3 signaling inhibition, Bcl-2 increase, caspase-3 and Bax downregulation (lncRNA LINC00346), miR-21-5p sponging, PNRC2 regulation, CRC progression inhibition (lncRNA OTUD6B-AS1), CRC development via miR-9-5p/STMN1 and IL-6R regulation (LINC01116), TPT1-mediated FAK and JAK-STAT3 signaling upregulation, CRC cell migration, miR-149-3p sponging, and CRC progression and metastasis (RNA TPT1-AS1 and RNA HOXA11-AS) (Huang et al., 2016b).

LINC00691 has regulated the JAK/STAT pathway and led to gastric cancer cell proliferation and invasion, which was confirmed using MKN-45 and HGC-27 cell lines and bioinformatics study, gene expression, luciferase gene reporter, Western blot, and *in vivo* (BALB/c nude mice) analyses (Liang et al., 2020). The inhibitor ruxolitinib could reverse the LINC00691 effects.

## Long non-coding RNAs and JAK/STAT regulation in colorectal cancer

It was observed that lncRNA RP11-468E2.5 exerted anticancer effects against CRC via apoptosis induction and precluding angiogenesis *in silico*, *in vitro* (tissue samples from 169 patients), and *in vivo*. lncRNA RP11-468E2.5 targeted the JAK/STAT signaling pathway via inhibition of STAT5 and STAT6. RP11-468E2.5 targeting using siRNA reversed the effects and inhibited apoptosis. Moreover, lncRNA LINC01116 has accelerated CRC progression via regulation of miR-9-5p/STMN1 and interleukin-6 receptor (IL-6R) (Bi et al., 2020b). LINC01116 exhibited a high expression level in CRC tissues, and its knockdown could hinder cancer progression. LINC01116 has also promoted cancer cell proliferation and migration (Xu et al., 2021). On the other hand, lncRNA AB073614 has exerted mesenchymal CRC cell tumorigenesis via JAK/STAT3 pathway regulation. It was highly expressed in CRC tissue, and its suppression in SW480 and HCT116 cells hindered the cell proliferation and invasion via expression of E-cadherin and occludin proteins. The phosphorylated STAT3 expression was also mitigated. lncRNA AB073614 has also decreased tumor growth, invasion, and metastasis; cell cycle arrest; and promotion of apoptosis. Other roles included regulation of EMT and Wnt-β catenin pathway (Liao et al., 2019; Liao et al., 2021; Yang et al., 2021).

It has been unraveled that lncRNA SUMO1P3 contributes to CRC cell proliferation via CPEB3 silencing and inhibition of apoptosis. Relevantly, CPEB3 affects the JAK/STAT pathway and decreases tumorigenesis of CRC cells (Fang et al., 2020). An *in silico* study using the R limma package and multivariate Cox regression unraveled that four lncRNAs, which were mostly associated with the JAK/STAT pathway, were also associated with stages II–III CRC. These included HAND2-AS1, MIR100HG, TRG-AS1, PCED1B-AS1, FAM30A, AL365361.1, AC090559.1, and LINC01094 (Brenner et al., 2014).

**FIGURE 3:**
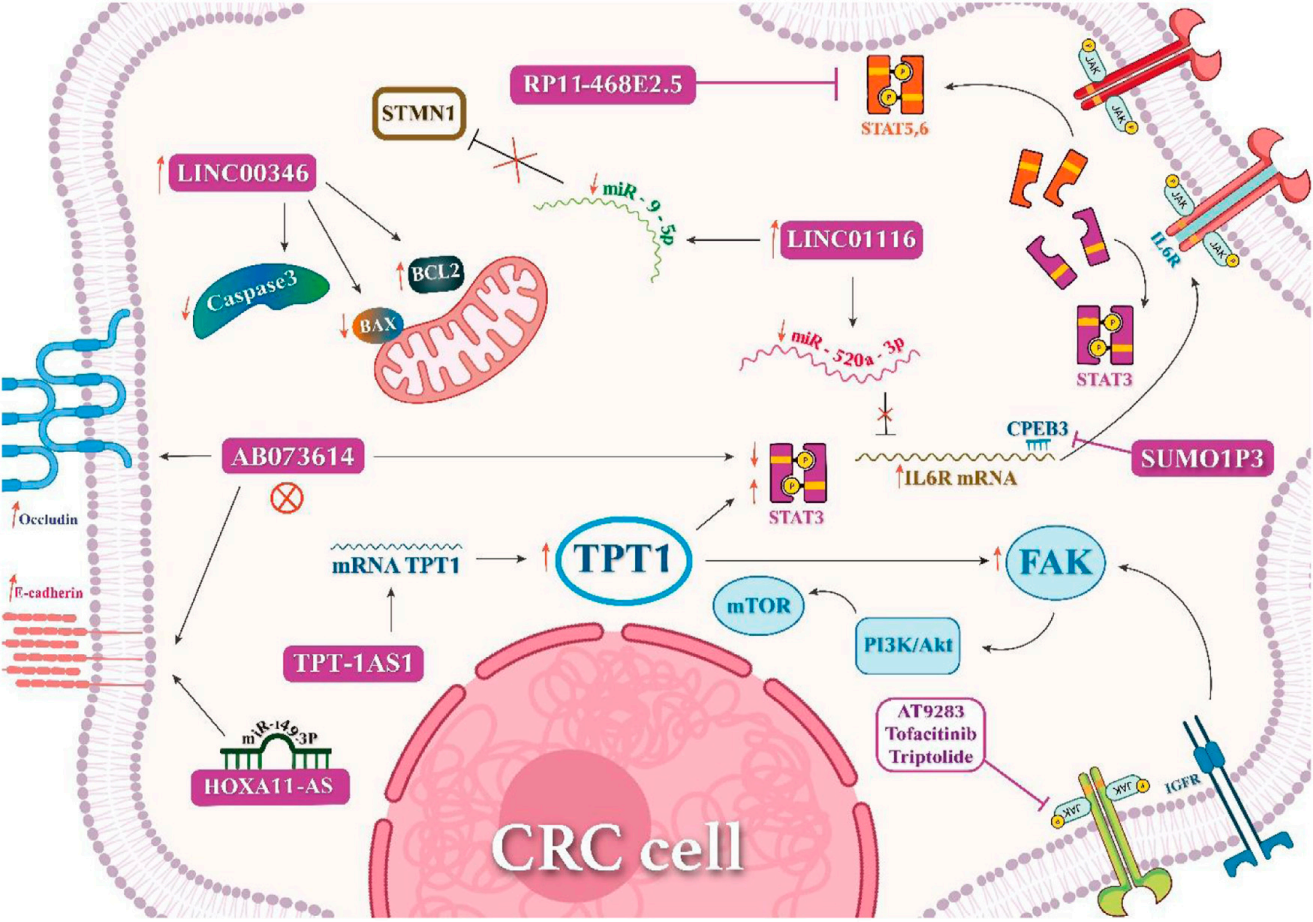
Various mechanisms of lncRNAs in CRC cell inhibition or proliferation.

lncRNA LINC00346 has been associated with CRC cell proliferation and invasion, Bcl-2 increase, and caspase-3 and Bax downregulation in HT29 and LoVo CRC cells. Its silencing was associated with apoptosis induction and inhibition of cancer progression via inhibiting the JAK/STAT signaling pathway. Interestingly, tofacitinib (JAK1 inhibitor) could reverse its cancer-promoting effects (Li and Wen, 2020). Moreover, triptolide, an inhibitor of JAK1 and phosphorylated STAT3, hindered the proliferation of CRC cells (Wang et al., 2009). Zhang L. et al. (2021) assessed 72 CRC and 36 adjacent normal tissues and revealed that lncRNA TPT1-AS1 facilitates CRC progression and metastasis through upregulation of JAK/STAT3 and FAK pathways by the expression of tumor protein translationally controlled 1 (TPT1). Additionally, *in vivo* findings revealed CRC cell proliferation by clone formation and tumor size/weight assay in nude mice. Chen et al. (2020)showed that the HOXA11-AS/miR-149-3p axis caused CRC cell proliferation and HCT116 cell migration. They also demonstrated that, with HOXA11-AS being its molecular sponge, miR-149-3p could lead to increased E-cadherin expression (Table 1; Figure 3). HOTAIR is another lncRNA overexpressed in CRC and promotes tumor growth and metastasis. HOTAIR interacts with the JAK-STAT pathway by binding to STAT3, leading to its activation and subsequent promotion of CRC cell proliferation and invasion (Liu et al., 2019). UCA1 is upregulated in CRC and activates the JAK-STAT pathway. UCA1 promotes CRC cell proliferation, migration, and invasion by enhancing STAT3 phosphorylation and nuclear translocation (Liu et al., 2021). GAS5 is downregulated in CRC and acts as a tumor suppressor. GAS5 inhibits the JAK-STAT pathway by interacting with JAK2 and preventing its phosphorylation. This leads to decreased STAT3 activation and suppression of CRC cell growth and invasion (Lin et al., 2022; Shakhpazyan et al., 2023).

**TABLE1:** lncRNA level changes and their mechanisms in CRC conditions.

lncRNA	Role in cancer	Study model/cells	Mechanism of action	Reference
ITIH4-AS1	Oncogene	*In vitro* */* *in vivo*	FUS-dependent JAK/STAT3 pathway activation	Huang et al. (2016b)
CASC2	Oncogene	Caco-2 and HT29	miR-18a sponging, JAK2/STAT3 activation, and proliferation	Huang et al. (2016b)
RP11-468E2.5	Inhibitor	*In vitro*	Apoptosis induction via STAT5 and STAT6	Jiang et al. (2019)
RP11-468E2.5	Inhibitor	GEO database, *in vitro* and *in vivo*	Apoptosis induction via targeting STAT5/6	Jiang et al. (2019)
lncRNA LINC01116	Oncogene	OUMS23, SW116, SW480, and LoVo cell lines	CRC development via miR-9-5p/STMN1 and IL-6R regulation	Liang et al. (2021b)
lncRNA AB073614	Inhibitor	*In vitro* (SW480 and HCT116 cells)	JAK/STAT3 pathway downregulation and expression of E-cadherin and occludin	Xue et al. (2018)
lncRNA LINC00346	Oncogene	HT29 and LoVo cells	Cell proliferation and inhibition of apoptosis, JAK1/STAT3 signaling inhibition, Bcl-2 increase, and Caspase-3 and Bax downregulation	Li and Wen (2020)
lncRNA OTUD6B-AS1	Inhibitor	SW620, SW480, HCT116, and RKO	miR-21-5p sponging, PNRC2 regulation, CRC progression inhibition	Cai et al. (2021)
LINC01116	Oncogene	Saos-2, U-2OS, and MG-63	CRC development via miR-9-5p/STMN1 and IL-6R regulation	Zhang et al. (2018)
RNA TPT1-AS1	Oncogene	SW620 cells, *in vivo*	TPT1-mediated FAK and JAK-STAT3 signaling upregulation and CRC cell migration	Zhang et al. (2021b)
RNA HOXA11-AS	Oncogene	HCT116 and SW480	miR-149-3p sponging and CRC progression and metastasis	Chen et al. (2020)
BC200	Oncogene	HCT116 and HT29	Regulation of JAK/STAT3 and Wnt/β-catenin pathways and CRC progression and metastasis	Wu et al. (2018)

lncRNA, long non-coding RNA; CASC2, cancer susceptibility 2; iPNRC2, proline-rich nuclear receptor coactivator 2; JAK1/STAT, Janus kinase/signal transducer and activator of transcription; miR, microRNA; CRC, colorectal cancer.

MALAT1 is upregulated in CRC and has been implicated in promoting tumor growth and metastasis. MALAT1 activates the JAK-STAT pathway by interacting with STAT3 and enhancing its phosphorylation, thereby contributing to CRC progression.

## Future prospects

In recent years, there has been a growing body of research that has shed light on the significant contribution of non-coding RNAs to the pathogenesis of different types of cancers (Wu J. et al., 2022; Gao et al., 2022; Liu et al., 2023; Xie et al., 2023). Specifically, lncRNAs and the JAK-STAT pathway have emerged as pivotal factors in the initiation and advancement of CRC (Li B. et al., 2022). The understanding of the biological functions and mechanisms of lncRNAs in cancer is essential for future studies to succeed in early diagnosis (as biomarkers) and achievement of appropriate therapeutic options (Dai et al., 2017; Xie et al., 2022). RNA sequencing revealed several lncRNAs as CRC biomarkers including CRCAL-1-4 (Yamada et al., 2018). The traits and pathophysiology of various cancers have been related to lncRNAs which can be considered “genetic debris” prognostic or diagnostic biomarkers. Their specific therapy is possible considering tissue-specific expression. Discovering lncRNA cross-talk and its effects on CRC progression is also helpful in understanding regulatory mechanisms. Consequently, these verification aspects contribute to the guide of inhibitory or activating drugs/compounds. As the detection of lncRNA expression is convenient in patients’ samples, they can be considered biomarkers of diagnosis or prognosis of cancers/disease type. The minimal side effects due to the lncRNA targeting in cancers (for oncogenic circuit blocking), lncRNA-based cancer therapy such as restricted expression is promising. Cancer-suppressing lncRNA expression can be enhanced using various approaches such as nanovectors. The knockout of lncRNAs using CRISPR-CAS9 is another approach for cancer therapy, which has reduced metastasis and increased the survival rate of mice (Zhuo et al., 2019). Targeting of the JAK/STAT pathway can lead to the activation of anti-tumor immune cells, and combination therapies are promising in this regard (Sabaawy et al., 2021). In addition, functional characterization of lncRNAs, identification of lncRNA biomarkers, therapeutic targeting of lncRNAs, elucidating the cross-talk between lncRNAs and the JAK-STAT pathway, and integration of lncRNAs and the JAK-STAT pathway into personalized medicine open new avenues in future.

## Conclusion

lncRNAs play substantial roles in the regulation of cancer-driving pathways, and their roles have been recently demonstrated in the field of oncology. lncRNAs can be considered for cancer theranostic aims. By unveiling the biological roles of lncRNAs in the regulation of epithelial cell proliferation and mechanisms of development to neoplasia and more, cancer diagnosis will face a promising future when lncRNA radiotracing technology comes to clinical use. The understanding of lncRNA interactions or their cross-talk is also important for efficient precluding of cancer development. Several lncRNAs play a role as oncogenic or tumor-suppressor agents via regulation of JAK/STAT signaling. Hence, the targeting of this pathway is crucial for efficacious CRC anti-tumor therapy.
